# Physiologically based pharmacokinetic models for systemic disposition of protein therapeutics in rabbits

**DOI:** 10.3389/fphar.2024.1427325

**Published:** 2024-08-28

**Authors:** Ravi Kumar Jairam, Maria Franz, Nina Hanke, Lars Kuepfer

**Affiliations:** ^1^ Institute for Systems Medicine with Focus on Organ Interaction, University Hospital RWTH Aachen, Aachen, Germany; ^2^ Translational Medicine & Clinical Pharmacology, Boehringer Ingelheim Pharma GmbH & Co. KG, Biberach, Germany

**Keywords:** PBPK, IgG, Fab, TMDD, rabbit pharmacokinetics

## Abstract

Physiologically based pharmacokinetic (PBPK) modelling is an important tool to predict drug disposition in the body. Rabbits play a pivotal role as a highly valued small animal model, particularly in the field of ocular therapeutics, where they serve as a crucial link between preclinical research and clinical applications. In this context, we have developed PBPK models designed specifically for rabbits, with a focus on accurately predicting the pharmacokinetic profiles of protein therapeutics following intravenous administration. Our goal was to comprehend the influence of key physiological factors on systemic disposition of antibodies and their functional derivatives. For the development of the systemic PBPK models, rabbit physiological factors such as gene expression, body weight, neonatal fragment crystallizable receptor (FcRn) binding, target binding, target concentrations, and target turnover rate were meticulously considered. Additionally, key protein parameters, encompassing hydrodynamic radius, binding kinetic constants (KD, k_off_), internal degradation of the protein-target complex, and renal clearance, were represented in the models. Our final rabbit models demonstrated a robust correlation between predicted and observed serum concentration-time profiles after single intravenous administration in rabbits, covering IgG, Fab, F(ab)_2_, Fc, and Fc fusion proteins from various publications. These pharmacokinetic simulations offer a promising platform for translating preclinical findings to clinical settings. The presented rabbit intravenous PBPK models lay an important foundation for more specific applications of protein therapeutics in ocular drug development.

## 1 Introduction

Over the past 2 decades, protein therapeutics such as monoclonal antibodies and their derivatives, have undergone a noteworthy evolution in treatment of a diverse range of diseases, including cancer, age-related macular degeneration, diabetic retinopathies and more. This advancement has made a substantial impact on patient health and wellbeing (Strohl, 2018). This success has been primarily driven by substantial advancements in the discovery, development, and approval of protein therapeutics. Prior to commencing preclinical animal experiments, a meticulous consideration of critical parameters is imperative to ensure the appropriateness and relevance of animal models in preclinical studies in pharmaceutical drug development. For protein therapeutics, such parameters involve amongst others antibody cross-reactivity, basic pharmacokinetics, and potential interactions of IgG with neonatal fragment crystallizable receptor (FcRn) in endosomes. These initial assessment ensures a first evaluation of the safety and efficacy of protein therapeutics in preclinical settings, and supports transition to clinical applications at later stages of pharmaceutical development (Loisel et al., 2007; Chen and Balthasar, 2012; Basu et al., 2020).

In the realm of preclinical drug development, rabbits have emerged as a vital link connecting preclinical models to clinical applications, particularly in the advancement of ocular therapeutics following intraocular routes of administration. This significance is attributed to the similarity in size between rabbit eyes and human eyes, setting them apart from other mammals (Basu et al., 2020). Additionally, due to the similarity in the nucleotide and amino acid sequences of our genes, the rabbit immune system demonstrates a closer resemblance to the human immune system compared to rodents (Mage et al., 2019). Before considering advanced applications of therapeutic proteins following ocular administration, it is essential to understand the pharmacokinetic mechanisms governing systemic disposition. This foundational knowledge serves as a prerequisite for unravelling the intricate complexities inherent in the mechanism of target binding. In this work, our focus was on establishing systemic physiologically based pharmacokinetic (PBPK) models for different antibodies and their fragments. These models not only hold promise for the prospective development of ocular models but also lay the foundation for modelling of other routes of administration in rabbits. PBPK modelling serves as a valuable mechanistic tool to understand and analyse drug pharmacokinetics. In pharmaceutical drug development, PBPK modelling has been used to simulate preclinical and clinical pharmacokinetics, for example, during drug-drug interactions, for *in vitro* to *in vivo* extrapolation or to compare different dosing schemes (Kuepfer et al., 2016; Wong and Chow, 2017; Mavroudis et al., 2018; Miller et al., 2019).

In the present work, PBPK modelling of intravenously administered biologics was utilized to assess the predictive potential of PBPK for protein therapeutics, specifically focusing on various antibodies and their fragments based on previously published preclinical pharmacokinetic (PK) studies in rabbits. In our study, protein PBPK models were developed that mechanistically incorporate the intricate dynamics of protein therapeutics. Starting from a standard protein PBPK model for non cross-reactive proteins (no target binding in rabbits), additional processes were stepwise introduced to describe target-mediated drug disposition (TMDD) (Niederalt et al., 2018). These extended models incorporate a comprehensive set of physicochemical/thermodynamic properties, including the hydrodynamic radius of the molecule, target interactions, as well as FcRn binding. The model development involved determining target concentrations, scrutinizing target synthesis rates, and analysing the degradation rate constants for the drug-target complexes.

We compiled diverse literature datasets for protein PK, encompassing an array of protein therapeutics. To fortify the reliability and predictive power of our models, a systematic model development process was performed, and model qualification involved a comprehensive comparison between our model simulations and the observed PK profiles documented in the literature. These models rely on the obtained serum concentration-time profiles of 10 protein therapeutics from rabbits reported in the literature. For each protein, potential relationships between estimated model parameters and *in vitro* assay results are investigated. The results underscore the ability of this proposed model-based framework, which mechanistically integrates all characteristics of antibodies determining their pharmacokinetics. The primary objective of this work is to predict intravenous PK profiles in rabbits, aiding in antibody screening in the early stages of development and facilitating extrapolation to humans.

## 2 Materials and methods

### 2.1 Experimental data

In our investigation, we used previously published rabbit PK profiles following intravenous bolus injections in naïve animals. The total serum concentration of antibodies and antibody fragments was determined using either enzyme-linked immunosorbent assay (ELISA) or electro-chemiluminescent assay in these studies. To digitize the reported data, we employed WebPlotDigitizer (https://automeris.io/WebPlotDigitizer/).

### 2.2 PBPK software

PK-Sim^®^ and MoBi^®^ from the Open System Pharmacology (OSP) Suite version 11.0 (https://www.open-systems-pharmacology.org, accessed on 01 October 2023) were used to simulate serum concentrations over time for monoclonal antibodies and their fragments. The protein PBPK model integrates detailed physiological and biochemical parameters to accurately simulate pharmacokinetics in various compartments representing multiple tissues and organs, and the disposition of therapeutic proteins. Unlike the standard PBPK model, it incorporates endosomal clearance and FcRn-binding within endosomal compartments, crucial for antibody recycling and extended half-life, and includes a TMDD process to model target binding. The model also features specific renal clearance mechanisms for proteins with lower molecular weight. Furthermore, it accounts for the movement of macromolecules through cellular pores via convection and diffusion, while excluding passive diffusion into cells, which is not relevant for large molecules like antibodies and their fragments.

Building on our earlier work by Niederalt et al. (2018) on therapeutic proteins, we used consistent terminology for antibodies and their fragments in this study. Initially, a PBPK model was developed in PK-Sim^®^, incorporating details such as average body weight from literature data, compound-specific information for protein therapeutics (including molecular weight, fraction unbound, solute radius, and equilibrium dissociation constant values for the neonatal Fc receptor in endosomal space), and additional renal clearance mechanisms for protein therapeutics with lower molecular weight (<69 kDa) (Zhao et al., 2012; Ovacik and Lin, 2018). A single intravenous bolus administration protocol was established, and the corresponding dose was considered in the model. Parameter identification was performed in PK-Sim^®^ for non cross-reactive molecules (Table 1). For cross-reactive protein therapeutics, rabbit gene expression data specific to the target of interest were used in the rabbit PBPK model (e.g., vascular endothelial growth factor (VEGF) data for anti-VEGF Fab, and a combination of VEGF and placental growth factor (PlGF) data for conbercept) (Meyer et al., 2012; Cordes and Rapp, 2023). The PBPK model was then extended in MoBi^®^ to include a TMDD process, capturing interactions and dynamics between the drug and its target. Target turnover, target binding and degradation of the protein-target complex were defined as represented by Equations (1–5). Parameter estimation was performed using a Monte Carlo algorithm for both cross-reactive and non cross-reactive cases, with estimated parameters detailed in the Supplementary Material for conbercept and anti-VEGF compounds. Data processing and visualization were conducted using the statistical programming language R (version 4.2.3).

**TABLE 1:** Standard parameters derived from our PBPK models for various monoclonal antibodies (mAbs) and their fragments in rabbit, mAbs; monoclonal antibodies, MW; molecular weight, FcRn; neonatal fragment crystallizable receptor, KD-FcRn; equilibrium dissociation constant of IgG with FcRn in rabbit, R_CL_; renal clearance.

mAbs	MW (kDa)	FcRn binding in rabbit	Target binding in rabbit	Solute radius (nm)	KD-FcRn (µmol/L)	R_CL_ (mL/min/kg)
Anti-gD IgG	150	Yes	No	4.13	2.52	‐
Anti-gD null IgG	150	Yes	No	4.13	13.3	‐
Anti-gD Fab	50	No	No	3.30	‐	0.70
Anti-gD F(ab)_2_	100	No	No	3.07	‐	0.05
Anti-gD Fc	50	Yes	No	2.40	1.30	0.04
Anti-VEGF Fab	50	No	Yes	2.40	‐	0.56
Anti-VEGF F(ab)_2_	100	No	Yes	3.70	‐	0.10
Obiltoxaximab (IgG)	148	Yes	No	4.13	7.95	‐
rabIgG (IgG)	150	Yes	No	4.86	3.40	‐
Conbercept (Fc fusion)	143	Yes	Yes	3.00	2.59	‐

### 2.3 Structure of the PBPK models

**FIGURE 1:**
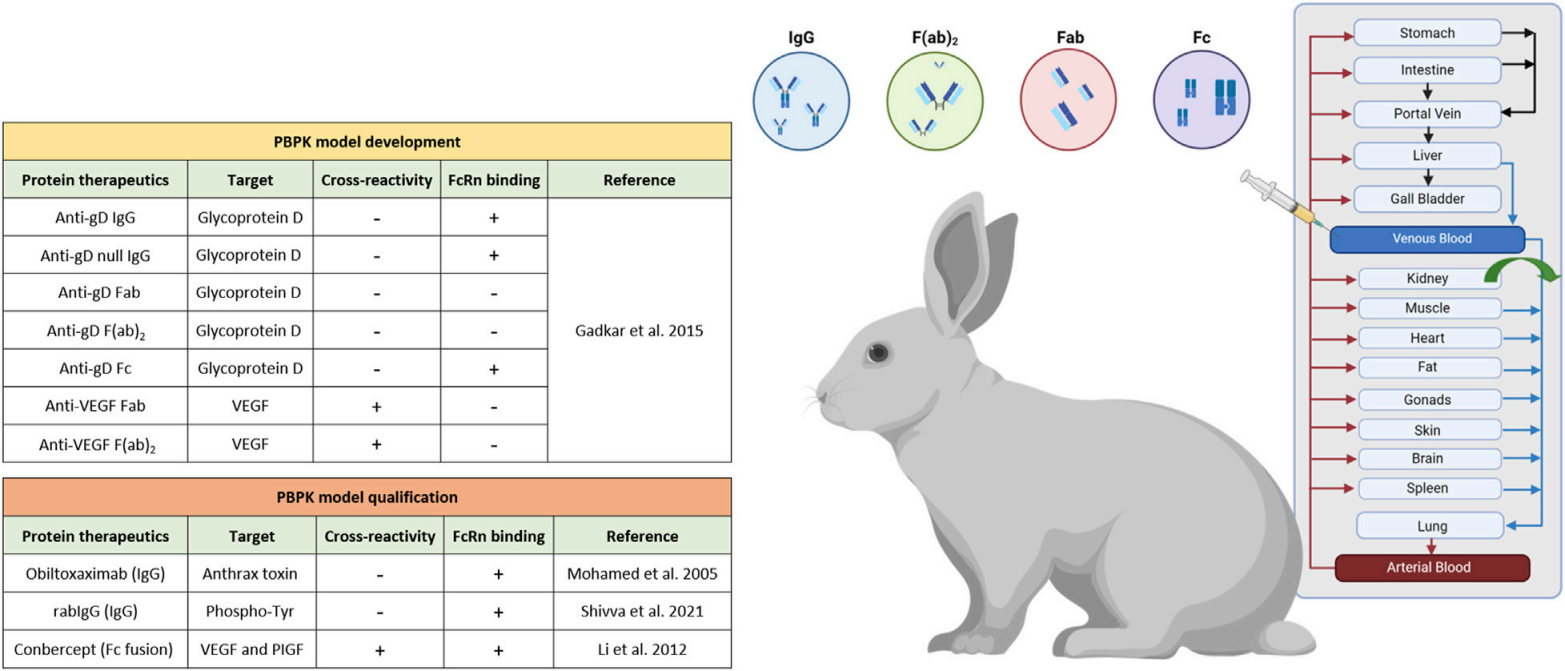
Schematic representation of the rabbit intravenous PBPK for a series of protein therapeutics within the PK-Sim^®^ software platform, created with Biorender.com.

Our PBPK model framework for monoclonal antibodies serves as a comprehensive and realistic representation of the physiological processes governing the pharmacokinetics of monoclonal antibodies (mAbs). All models, as depicted in Figure 1, provide a mechanistic description of distribution, metabolism, and elimination of protein therapeutics, involving various physiological factors and processes. The protein PBPK model framework incorporates the FcRn mediated endocytic salvage pathway, which is crucial to describe IgG recirculation (Niederalt et al., 2018). This pathway occurring within endosomes, is essential for maintaining the homeostasis and prolonged half-life of IgG antibodies in the body. More details on the relevant reactions and equations for target and FcRn binding can be found elsewhere (De Witte et al., 2023). In the context of mAbs or Fc fusion molecules pharmacokinetics, FcRn binding emerges as the primary determinant for extended serum half-life (Raghavan et al., 1995; Liu, 2018; Niederalt et al., 2018; Mackness et al., 2019).

For non cross-reactive proteins in rabbits, the original protein PBPK model in PK-Sim^®^ was used. However, to account for TMDD of cross-reactive proteins, we expanded the model using MoBi^®^ to incorporate target engagement as well as target synthesis and degradation (Meyer et al., 2012; Cordes and Rapp, 2023), as delineated by the mathematical equations outlined below:

$$k_{on}=\frac{k_{off}}{KD}$$ (1)

$$\frac{dD}{dt}=-\;k_{on}\;•\;T\;•\;D+k_{off}\;•\;DT$$ (2)

$$R_{synthesis}=k_{to}•\;T_{ss}$$ (3)

$$\frac{dT}{dt}=R_{synthesis}-T\;•\;k_{to}\;-\;k_{on}\;•\;T\;•\;D+k_{off}\;•\;DT$$ (4)

$$\frac{dDT}{dt}=k_{on}\;•\;T\;•\;D\;-\;k_{off}\;•\;DT\;-\;k_{\deg}•\;DT$$ (5)

Where k_on_ and k_off_ are drug target association and dissociation rate constants, respectively, KD represents the equilibrium dissociation constant, D is the drug concentration, T is the target concentration, DT is the drug target complex concentration, R_synthesis_ is the synthesis rate of the target, T_ss_ is the steady state concentration of the target, k_to_ is the target turnover rate constant, and k_deg_ is the drug target complex degradation rate constant.

## 3 Results

### 3.1 Literature search on currently available intravenous PK studies in rabbits for antibodies and their fragments

We started our analysis with an exhaustive literature research of previously published PK studies of antibodies and their fragments in healthy rabbits. Altogether, the search yielded a total of 10 protein therapeutics research works, which capture the diversity of therapeutic antibodies with intravenous PK data specifically in rabbits (Figure 1). Of note, mAbs that followed different formulation approaches (e.g., microspheres, liposomes, etc.) or novel drug delivery methods were intentionally omitted from the analysis. Furthermore, our model development significantly benefitted from the comprehensive dataset provided by Gadkar et al., encompassing both cross-reactive and non cross-reactive antibodies and their fragments (Gadkar et al., 2015). In parallel, our model development considered antibody fragments such as Fab and F(ab)_2_, which specifically exclude the fraction crystallizable region (Fc) portion, so that for these molecular species any considerations related to FcRn binding could be neglected. Renal clearance was incorporated for proteins with lower molecular weight (<69 kDa) (Zhao et al., 2012; Ovacik and Lin, 2018), as it plays a significant role in the clearance of these smaller proteins from the body, in contrast to typical mAbs (∼150 kDa) where renal clearance does not play a role. The differences in molecular weight between the smaller proteins and typical mAbs lead to different pharmacokinetic behaviors and clearance mechanisms. However, it is worth highlighting that parameters associated with target engagement and TMDD depend on target specific values such as the target concentration, the equilibrium dissociation constant (KD), dissociation rate constant (k_off_), target turnover rate constant (k_to_) and internal degradation rate constant of the mAb target complex (k_deg_). These values exhibit variation depending on the specificity of the targeted molecule. The interactions between the drug and its target play a pivotal role in determining the dynamics of drug distribution, metabolism, and elimination. By carefully considering these factors and parameters, our models have been tailored to provide accurate predictions and insights. For rigorous model qualification, we employed data on protein therapeutics selected from various publications, carefully curated, and sorted based on their verified cross-reactivity in rabbits (Figure 1; Table 1). The final dataset comprises 10 protein therapeutics including IgG, Fab, F(ab)_2_, Fc and Fc fusion proteins and provides valuable insights into how different protein therapeutics interact with the rabbit’s physiology, which is essential for the development and optimization of these proteins for potential therapeutic use.

The primary dataset used for our PBPK model development originates from the work of Gadkar et al. (2015). This study, was focused on anti-glycoprotein D (Anti-gD), a non-modified IgG, and its corresponding fragments (Fab, F(ab)_2_, and Fc), derived from a singular source, targeted to glycoprotein D on the viral envelope. Notably, this antibody (IgG) or its fragments, do not typically undergo TMDD. Since the specific target of the antibodies is glycoprotein D (present on the HIV viral envelope), is missing in healthy rabbits, TMDD does not occur here. Furthermore, a separate set of antibody fragments targeting vascular endothelial growth factor (VEGF) was investigated, which undergo TMDD. This set of data was instrumental in informing model development since different physiological processes and their corresponding parameters could be identified in a step-by-step manner.

**FIGURE 2:**
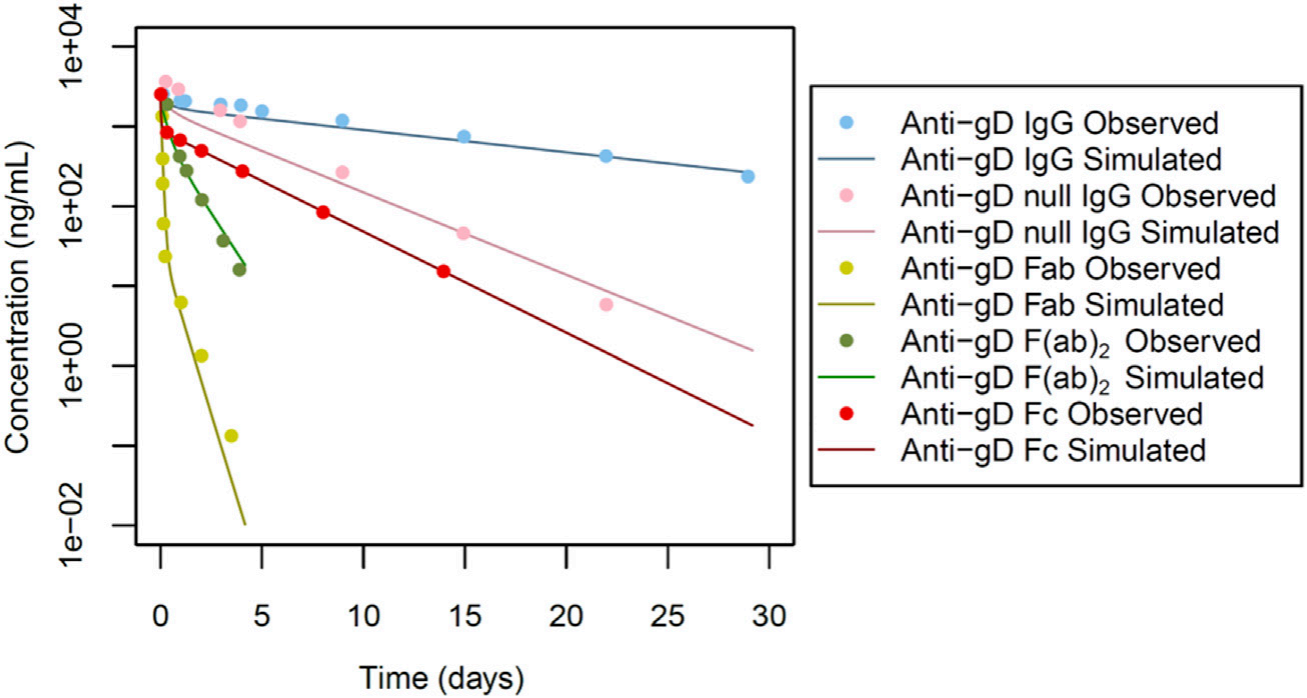
Simulated versus observed serum concentration-time profiles for non cross-reactive antibodies and their fragments in rabbit (data from Gadkar et al., 2015).

**FIGURE 3:**
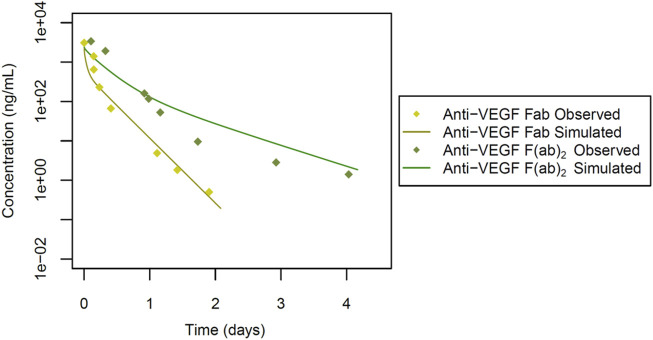
Simulated versus observed serum concentration-time profiles for cross-reactive antibody fragments in rabbit (data from Gadkar et al., 2015).

The study design by Gadkar et al. involved the administration of intravenous doses of antibodies or antibody fragments to male New Zealand white (NZW) rabbits (n = 3) at a dose of 0.5 mg. The pharmacokinetic profiles were evaluated over a 28-day period (Figures 2, 3), providing valuable insights into the disposition of the administered antibodies and fragments following intravenous dosing in this specific experimental context. Specific administration protocols were considered in each of the PBPK models in this study.

**FIGURE 4:**
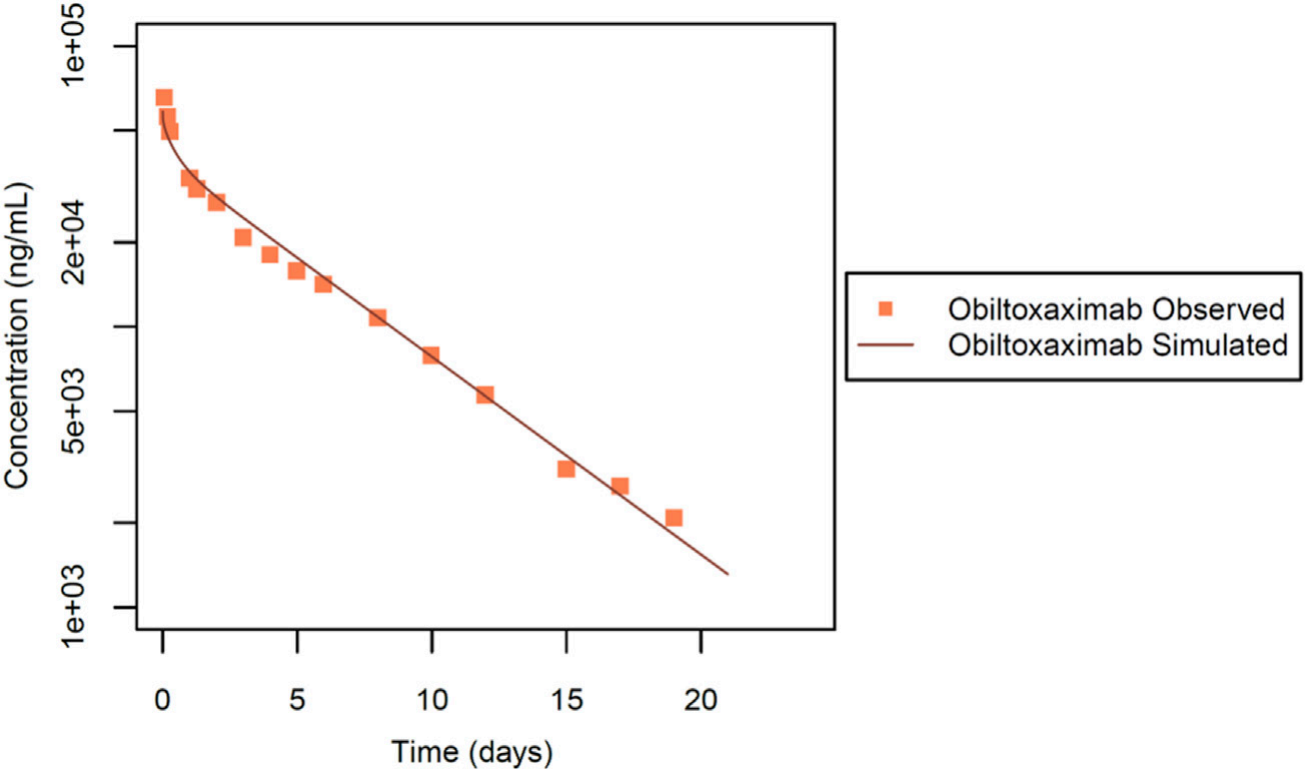
Simulated versus observed serum concentration-time profiles for obiltoxaximab IgG in rabbit (data from Mohamed et al., 2005).

The second dataset used for IgG intravenous PBPK model evaluation was from Mohamed et al. (Mohamed et al., 2005). In this study, obiltoxaximab (ETI-204), designed as a treatment targeting anthrax toxin produced by *Bacillus anthracis*, a Gram-negative bacterium, was considered. Notably, the authors assessed the PK profile of obiltoxaximab in healthy rabbits, ensuring that these antibodies did not undergo TMDD. The experimental design involved NZW rabbits (n = 3). In this dataset (Figure 4), the PK profile of the antibody was assessed following a single intravenous dose at 10 mg evaluated up to 21 days post injection.

**FIGURE 5:**
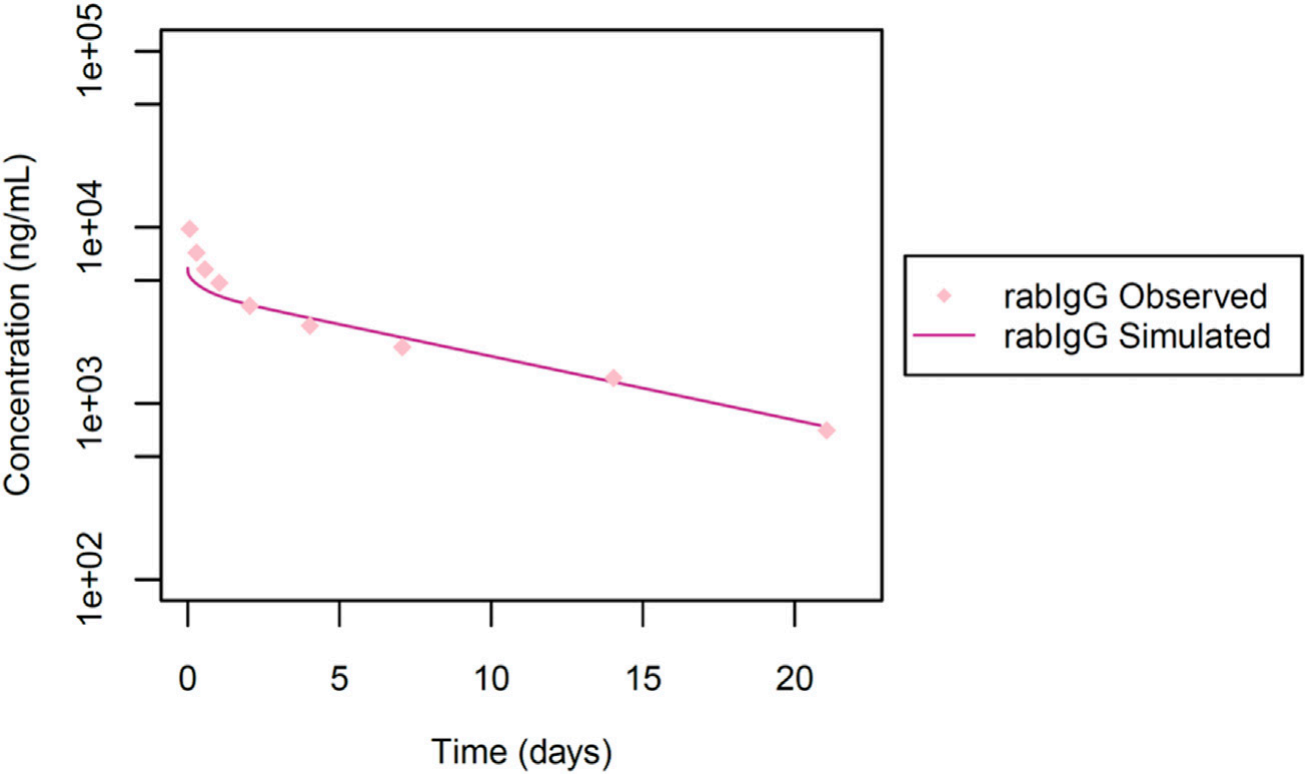
Simulated versus observed serum concentration-time profiles for rabIgG in rabbit (data from Shivva et al., 2021).

Additionally, we also made use of the dataset from Shivva et al. (2021), for model qualification, to investigate the systemic exposure of a rabbit antibody (rabIgG) following an intravenous dose of 1 mg. This IgG targets phosphorylated tyrosine (phospho-Tyr), and is not anticipated to undergo TMDD as it is targeted to intracellular proteins and the IgG is not expected to penetrate cells. In this study (Figure 5) the pharmacokinetic evaluation was conducted in NZW rabbits (n = 12) up to 21 days post-dose.

**FIGURE 6:**
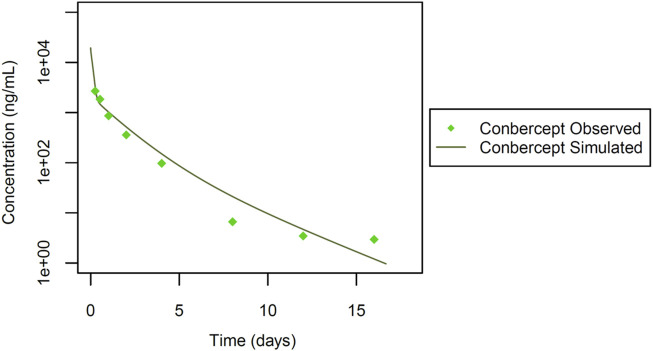
Simulated versus observed serum concentration-time profiles for conbercept Fc fusion protein in rabbit (data from Li et al., 2012).

Finally, we incorporated data from a study by Li et al. (2012). In this study (Figure 6), systemic pharmacokinetics of the fusion protein conbercept (KH902) were assessed following intravenous bolus dosing in chinchilla rabbits (n = 6) at a dose of 3 mg. Conbercept acts as a receptor decoy with potent affinity for vascular endothelial growth factor (VEGF) and placental growth factor (PlGF) (Wang et al., 2013). Wang et al. explored conbercept’s cross-reactivity in rabbits through immunohistochemistry (Wang et al., 2013), which implies that conbercept can engage in TMDD due to its dual binding sites. This insight into the pharmacokinetic behavior of conbercept is essential, particularly in the context of its interaction with VEGF and PlGF in rabbits, given its dual binding capabilities. Additionally, the interaction of conbercept’s Fc portion with the FcRn receptor adds further complexity to its pharmacokinetics. These inclusions enrich our dataset diversity and complexity, incorporating a different therapeutic agent and its specific pharmacokinetic characteristics, allowing for a more comprehensive and robust development and qualification of the rabbit PBPK model as detailed in Figure 1.

### 3.2 Development of intravenous rabbit PBPK models for Anti-gD antibody and its fragments

As outlined above, the dataset by Gadkar et al., represents the starting point for our model development (Gadkar et al., 2015). This dataset enabled the simulation of concentration-time profiles in serum for non cross-reactive anti-glycoprotein (Anti-gD) antibodies, excluding TMDD. However, for cross-reactive anti-VEGF antibodies fragments, TMDD was incorporated.

For anti-gD IgG, weighing approximately 150 kDa, we focused on a key factor in the model: the equilibrium dissociation constant (KD) of IgG with rabbit FcRn (KD-FcRn), indicating IgG’s binding affinity to rabbit FcRn. Due to a lack of reported values, KD-FcRn was empirically adjusted to align with the observed data (Figure 2) using starting values derived from humans (Basu et al., 2020). The optimal fitting values were determined as KD-FcRn = 2.52 μmol/L and, similarly, the hydrodynamic radius of the solute (Rh) was fitted, using the value calculated from Hutton-Smith et al. (Hutton-Smith et al., 2016) as starting value, resulting in a fitted value for Rh = 4.13 nm (Table 1). However, when examining anti-gD null IgG, a variant engineered to restrict binding to FcRn, the KD-FcRn fitting parameter was determined to be 13.26 μmol/L. This difference is due to its decreased ability to bind FcRn, as reported by Gadkar et al. (Gadkar et al., 2015). It is worth noting that despite these differences in binding affinity, the Rh value remained constant due to consistent molecular weight (Figure 2 for anti-gD IgG and anti-gD null IgG).

Next in our model-building efforts we analyzed non cross-reactive anti-gD Fabs in rabbits, considering the Rh and renal clearance (R_CL_) as the most impactful parameters. Given that F (ab)_2_, a dimer linked by single or double di-sulfide bonds, tends to break down to two Fab monomers *in vivo* with a size of approximately 50 kDa each, we deemed the Fabs renal clearance values to be critical parameters. The R_CL_ was determined to be 0.70 mL/min/kg for a monomer with a molecular weight of 50 kDa for anti-gD Fab. Initial values were derived from reported rabbit data (Li and Shah, 2019), and were subsequently fine-tuned to better align with the observed profile (Figure 2 for anti-gD Fab). Furthermore, the Rh value underwent adjustment through parameter optimization, resulting in a refined value of 3.30 nm (Table 1).

Additionally, the anti-gD F(ab)_2_, a dimer with a molecular weight of ∼100 kDa was considered. The fragments of this protein are linked via double di-sulphide bonds with a tendency to break into monomers *in vivo*. The simulated profiles, as depicted in Figure 2 for anti-gD F(ab)_2_, applied an īkestimated R_CL_ value of 0.05 mL/min/kg. The Rh value was assumed and fixed at 3.07 nm based on estimates from Hutton-Smith et al. (Hutton-Smith et al., 2016) (Table 1).

Next, the anti-gD Fc protein with a molecular weight of ∼50 kDa was considered (Figure 1). The solute radius Rh was set at 2.40 nm, aligning with the calculated Rh values based on molecular weight from Hutton-Smith et al. (2016). Other parameters, including a KD-FcRn value of 1.30 μmol/L and R_CL_ of 0.04 mL/min/kg (Table 1) were estimated. The resulting simulated profiles for anti-gD Fc are presented in Figure 2.

### 3.3 Development of intravenous rabbit PBPK models for Anti-VEGF antibody fragments

To next extend the PBPK model to cross-reactive Fab fragments, we considered specific characteristics associated with their interaction with targets in rabbits. Due to the absence of the Fc region, the KD-FcRn parameter was omitted from these models. However, for antibody fragments like the monomer Fab and dimer F(ab)_2_, we incorporated TMDD, considering their cross-reactivity in rabbits. We started with the cross-reactive anti-VEGF Fab, a monomer with a molecular weight of 50 kDa (Table 1; Figure 3). The Rh was set to 2.40 nm based on the calculated value from Hutton-Smith (Hutton-Smith et al., 2016), while the parameters governing TMDD with VEGF were estimated: KD = 20 pmol/L and k_off_ = 7.30E-6 1/sec, with initial values derived from reported ranibizumab data (Papadopoulos et al., 2012) based on their similar molecular weight. The target VEGF concentration in rabbits was estimated to be 1.15E-7 μmol/L, k_to_ = 2.78E-4 1/min, and k_deg_ = 1.13E-7 1/min, based on starting values derived from humans (Basu et al., 2020), R_CL_ was estimated at 0.56 mL/min/kg. The simulations featuring anti-VEGF Fab compared to observed values are shown in Figure 3. Model parameter values are documented in Supplementary Figure S1.

For the anti-VEGF F(ab)_2_ dimer, with a molecular weight of 100 kDa (Table 1; Figure 3), the Rh was estimated to be 3.70 nm. Additionally, values for TMDD were optimized (KD = 0.49 pmol/L and k_off_ = 0.19 1/sec) using initial values derived from reported data on a VEGF trap with similar molecular weight (Papadopoulos et al., 2012). The VEGF concentration and k_to_ were applied, along with the estimated parameters k_deg_ = 0.98 1/min, and R_CL_ = 0.10 mL/min/kg (Table 1). The simulations featuring anti-VEGF F(ab)_2_ are depicted in Figure 3. Model parameter values are documented in Supplementary Figure S2.

### 3.4 Qualification of a rabbit intravenous PBPK model for obiltoxaximab

To validate our model platform, we analysed the PK data of obiltoxaximab in serum (Mohamed et al., 2005), for a 10 mg dose. Obiltoxaximab is an IgG with a molecular weight of 148 kDa and lacks TMDD. After estimation of only one parameter, the simulation results showed excellent agreement with the available experimental data (Figure 4). The KD-FcRn value was estimated to be 7.95 μmol/L. It is noteworthy that the Rh value was held constant, consistent with the molecular weight and previous simulations conducted for IgGs (Table 1).

### 3.5 Qualification of a rabbit intravenous PBPK model for rabIgG

Next, we utilized data from Shivva et al. for the simulation of the rabbit immunoglobulin G (rabIgG) with a molecular weight of 150 kDa. No TMDD was documented (Shivva et al., 2021). In this context, a reported Rh value of 4.86 nm was applied and the KD-FcRn value was determined to be 3.40 μmol/L (Table 1; Figure 5). However, our attempts to also simulate rabFab using data from the same publication were not successful, possibly attributed to challenges associated with the stability of the Fab fragment (simulations not presented).

### 3.6 Qualification of a rabbit intravenous PBPK model for conbercept

Finally, we simulated the Fc fusion protein conbercept (Li et al., 2012). Conbercept (KH902), with a reported molecular weight of 143 kDa, was administered as a single intravenous bolus at 3.0 mg. Studies evaluating the cross-reactivity of conbercept in rabbits, through immunohistochemistry and *in vitro* surface plasmon resonance (SPR) analysis, indicated dual target binding of conbercept to VEGF and PlGF (Wang et al., 2013; Wu et al., 2013; Wu et al., 2017). Similar concentrations of VEGF and PlGF have been reported in healthy humans (Zhou et al., 2014). It was hence assumed, that the PlGF reference concentration is identical to that of VEGF as identified from the rabbit PBPK model for anti-VEGF Fab shown above.

Additionally, KD-FcRn was fitted to 2.59 μmol/L, and the Rh value of conbercept was estimated at 3.00 nm. This estimation was derived from initial values calculated for bevacizumab with a molecular weight close to that of conbercept, as outlined by Hutton-Smith et al. (2016). For VEGF, the TMDD parameters were estimated with KD-VEGF = 1.53 pmol/L, k_off_-VEGF = 0.02 1/sec and k_deg_ = 2.22E-4 1/min, k_to_-VEGF was retained at the same value as in the previous simulations. Similarly, for PlGF, TMDD parameters were estimated with KD-PlGF = 1.87E-3 pmol/L, k_off_ -PlGF = 0.02 1/sec and k_deg_ = 615 1/min, k_to_-PlGF = 2.03E+04 1/min ( Supplementary Figure 3). After parameter identification, the PBPK model for conbercept showed good agreement with the experimental data (Table 1; Figure 6). The foundational parameters utilized in constructing systemic PBPK models along with reported values for both monoclonal antibodies (mAbs) and their fragments in rabbits are summarized in Table 1.

## 4 Discussion

The use of antibodies and their fragments for the treatment of a variety of diseases has been established over the past few decades (Lu et al., 2020). Not only full antibodies but also functional derivatives are gaining interest in the treatment of an array of diseases like cancer, rheumatoid arthritis, ocular complications, etc. (Keizer et al., 2010; Del Amo et al., 2017; Arlotta and Owen, 2019). The ability to accurately predict human PK from preclinical data is invaluable for the effective design and efficient conduct of first-in-human trials. Several approaches for human PK prediction using traditional allometric scaling have been broadly discussed in the literature for small molecules (Mahmood, 2007; Jairam et al., 2019a; Jairam et al., 2019b), however, there is only limited literature for large molecules (Mahmood, 2009; Malik and Edginton, 2019). PBPK modelling offers mechanistic insights into the distribution, metabolism, and elimination of drugs at organ level. In the future, these models will be pivotal in advancing the mechanistic translation of preclinical pharmacokinetic data pertaining to antibodies and their fragments into clinical practice.

In this research, our primary focus was on crafting intravenous PBPK models customized specifically for rabbits, a species extensively utilized in ocular drug development studies (Del Amo and Urtti, 2015). This work presents a notable extension to previously reported intravenous PBPK models which were limited to mAbs only (Bussing and Shah, 2020). Importantly, our PBPK platform encompasses both non cross-reactive and cross-reactive proteins, incorporating a comprehensive range of entities including antibody and their fragments such as Fabs, F(ab)_2_ and Fc. Since we were building on the concepts from our earlier work by Niederalt et al. (Niederalt et al., 2018) on therapeutic proteins, we wanted to use the same terminology for antibodies and their fragments in this paper. Our overarching objective was to establish a robust PBPK modelling platform capable of accurately describing the intravenous pharmacokinetics for protein therapeutics in rabbits. To achieve this, we meticulously validated the models using data sources from various publications. This rigorous stepwise qualification process, along with carefully selected literature data, ensured the reliability and broad applicability of the models, empowering it to effectively describe the intricate PK profiles associated with diverse protein therapeutics in rabbits.

Our proposed models accommodate a spectrum of antibodies and their fragments ranging from 50–150 kDa. The model development incorporated expression levels of targets such as VEGF and/or PlGF specifically in healthy rabbits, as well as binding dynamics to the FcRn receptor and kinetic constants related to protein interactions with the target, catabolic degradation, and turnover of drug-target complexes. Our models are also validated to describe proteins with lower molecular weight and their elimination through renal clearance (Ovacik and Lin, 2018). Steady state target concentrations of VEGF and PIGF, were derived from gene expression databases, which provide relative expression levels across various organs and tissue compartments and include whole-body gene expression data for healthy rabbits (Meyer et al., 2012; Cordes and Rapp, 2023).

The identification of the KD value for FcRn was conducted considering the starting value reported for bevacizumab in humans (Basu et al., 2020). This step was crucial for simulations involving monoclonal antibodies (mAbs) inclusive of the Fc part, within the molecular weight range of ∼150 kDa. From our comprehensive findings, the estimated average value for IgGs was KD-FcRn = 4.62 μmol/L ( Supplementary Table 1) for simulating the binding interaction between rabbit FcRn and humanized IgG in rabbits. This value is about 5-fold higher than the value for human FcRn estimated from a human bevacizumab PBPK model (Basu et al., 2020). This is consistent with *in vitro* studies reporting higher KD-FcRn values in rabbits, signifying lower binding affinity in rabbits than in humans (Szikora et al., 2017). The hydrodynamic radius (Rh) estimated for various IgGs with a molecular weight of approximately 150 kDa closely corresponds to the reported value of bevacizumab (Hutton-Smith et al., 2016) which shares a similar molecular weight. Our study yielded an average Rh value of 4.15 nm for IgG. Likewise, the hydrodynamic radius estimates for different Fabs, each with a molecular weight around 50 kDa, align with those reported for ranibizumab (Hutton-Smith et al., 2016) resulting in an estimated average value of 2.70 nm for the various Fab monomers and 3.39 nm for F(ab)_2_ dimers as shown in Supplementary Table 1.

Renal clearance occurs in a size-dependent manner, and protein therapeutics of molecular weight <69 kDa are primarily cleared through renal mechanisms, predominantly via glomerular filtration (Zhao et al., 2012). Consequently, for antibody fragments, the baseline value of renal clearance (R_CL_) was determined based on the observed preclinical data (Li and Shah, 2019), yielding estimated averages of 0.63 mL/min/kg for Fabs and 0.08 mL/min/kg for F(ab)_2_, which is around 5.6- to 14-fold lower than for monomer Fabs (Supplementary Table 1). This suggests that dimers exhibit a slower renal clearance than monomers, attributed to their size. However, we noted around 18-fold reduction in renal clearance (R_CL_) for the Fc fragment compared to anti-gD Fab, despite it being a 50 kDa protein. This observation raises the hypothesis that during renal clearance, Fab fragments, showing higher R_CL_, undergo both reabsorption and secretion within the renal tubules. In contrast, Fc fragments, showing lower R_CL_, were reported to predominantly undergo reabsorption with minimal secretion, possibly facilitated by their binding to FcRn (Ovacik and Lin, 2018; Gburek et al., 2021).

In a subsequent step, we integrated TMDD to model the anti-VEGF Fab monomer, dimer and the Fc fusion protein conbercept, considering their cross-reactivity in rabbits. *In vitro* values for kinetic constants from ranibizumab and VEGF trap were employed as initial estimates (Papadopoulos et al., 2012), based on their similarities in molecular weight and target binding. Throughout our simulations, we maintained consistent estimated VEGF reference concentrations and k_to_ values during the development of our models for anti-VEGF Fab, anti-VEGF F(ab)_2_ and conbercept. However, binding kinetic constants (KD, k_off_) and the internal degradation of complexes (k_deg_) varied across different VEGF protein therapeutics (additional information can be found in Supplementary Figures 1–3). This variability in drug-target complex related parameter values may indicate that they are either caused by size/shape/amino-acid-sequence differences, or have not been conclusively identified yet, potentially serving as a limitation in our model building. Additionally, the anti-VEGF F(ab)_2_ does not align well with the observed data (Figure 3), likely due to the different stability pattern of the dimeric molecule, where monomers linked by a single or double di-sulphide bond tend to cleave differently *in vivo* (Gadkar et al., 2015). For rabFab, our simulation attempts were not successful, which could be attributed either to increased aggregation or lower stability of the Fab fragment. The intrinsic instability of Fab fragments can alter the pharmacokinetics, making accurate modelling challenging. Moreover, the distinct pharmacokinetic behaviour of Fab fragments compared to full-length antibodies may necessitate more refined modelling approaches to capture their unique distribution and elimination patterns. For rabIgG, we were unable to achieve a good fit at earlier time points (Figure 5), likely due to the cluttering of observed data in the initial phase. Likewise, the usage of a small number of animals (n = 3) as reported data may introduce variability and limit the robustness of our findings, highlighting the need for larger sample sizes in future studies.

Our comprehensive study outlines established standard parameter values for protein therapeutics, derived through retrospective analysis, which are summarized in Table 1. This compilation serves as a sturdy foundation for future simulations of antibodies and their fragments in rabbits, providing crucial insights for predicting the *in vivo* behaviour of protein therapeutics.

In conclusion, our study focused on developing PBPK models tailored to rabbits in the context of protein therapeutics. The carefully developed and evaluated models cover a broad spectrum of antibodies and antibody fragments, incorporating gene expression data, binding dynamics, and hydrodynamic properties. The identified KD-FcRn values and renal clearance parameters are in good agreement with the various literature PK data sets used. Additionally, TMDD was mechanistically incorporated. The comprehensive PBPK models developed for antibodies and their fragments in this study provide a robust platform for further studies of protein therapeutics in rabbits.

## Data Availability

The original contributions presented in the study are included in the article/Supplementary Material, further inquiries can be directed to the corresponding author.
