# Supplementary material for: Physiologically based pharmacokinetic models for systemic disposition of protein therapeutics in rabbits
Source: Front Pharmacol. 2024 Aug 28;15:1427325. doi: 10.3389/fphar.2024.1427325 (PMC11387799; doi:10.3389/fphar.2024.1427325)
Supplement: Supplementary file 1 [file DataSheet1.docx]

Supplementary Material

Physiologically based pharmacokinetic models for systemic disposition of protein therapeutics in rabbits

Ravi Kumar Jairam^1^, Maria Franz^2^, Nina Hanke^2^, Lars Kuepfer^1*^

^1^Institute for Systems Medicine with Focus on Organ Interaction, University Hospital RWTH Aachen, Aachen, Germany

^2^Translational Medicine & Clinical Pharmacology, Boehringer Ingelheim Pharma GmbH & Co. KG, Biberach, Germany

*** Correspondence:**Lars Kuepfer, Institute for Systems Medicine with Focus on Organ Interaction, University Hospital RWTH Aachen, Aachen, Germany.

Email: lkuepfer@ukaachen.de

# Supplementary Figures and Tables

## Supplementary Tables

| **mAbs** | **MW**  **(kDa)** | **FcRn binding in rabbit** | **Target binding in rabbit** | **Solute radius**  **(nm)** | **KD-FcRn**  **(µmol/L)** | **R_CL_**  **(mL/min/kg)** |
| --- | --- | --- | --- | --- | --- | --- |
| Anti-gD IgG | 150 | Yes | No | 4.13 | 2.52 |  |
| Anti-gD null IgG | 150 | Yes | No | 4.13 | 13.3^#^ |  |
| Obiltoxaximab (IgG) | 148 | Yes | No | 4.13 | 7.95 |  |
| Bevacizumab (IgG) | 150 | Yes | Yes | 3.51* |  |  |
| rabIgG (IgG) | 150 | Yes | No | 4.86 | 3.40 |  |
| **Average ± SD** |  |  |  | **4.15 ± 0.31** | **4.62 ± 2.91** |  |
| Anti-gD Fab | 50 | No | No | 3.30 |  | 0.70 |
| Anti-VEGF Fab | 50 | No | Yes | 2.40 |  | 0.56 |
| Ranibizumab (Fab) | 50 | No | Yes | 2.40* |  |  |
| **Average ± SD** |  |  |  | **2.70 ± 0.52** |  | **0.63** |
| Anti-gD F(ab)_2_ | 100 | No | No | 3.07 |  | 0.05 |
| Anti-VEGF F(ab)_2_ | 100 | No | Yes | 3.70 |  | 0.10 |
| **Average ± SD** |  |  |  | **3.39** |  | **0.08** |

**Supplementary Table 1.** Standard PBPK model parameters for monoclonal antibodies (mAbs) and their fragments in rabbit with average ± SD values, mAbs; monoclonal antibodies, MW; molecular weight, FcRn; neonatal fragment crystallizable receptor, KD-FcRn; equilibrium dissociation constant of IgG with FcRn in rabbit, R_CL_; renal clearance, * data obtained from Hutton-Smith et al.(Hutton-Smith et al., 2016), ^#^ excluded from calculation

## Supplementary Figures


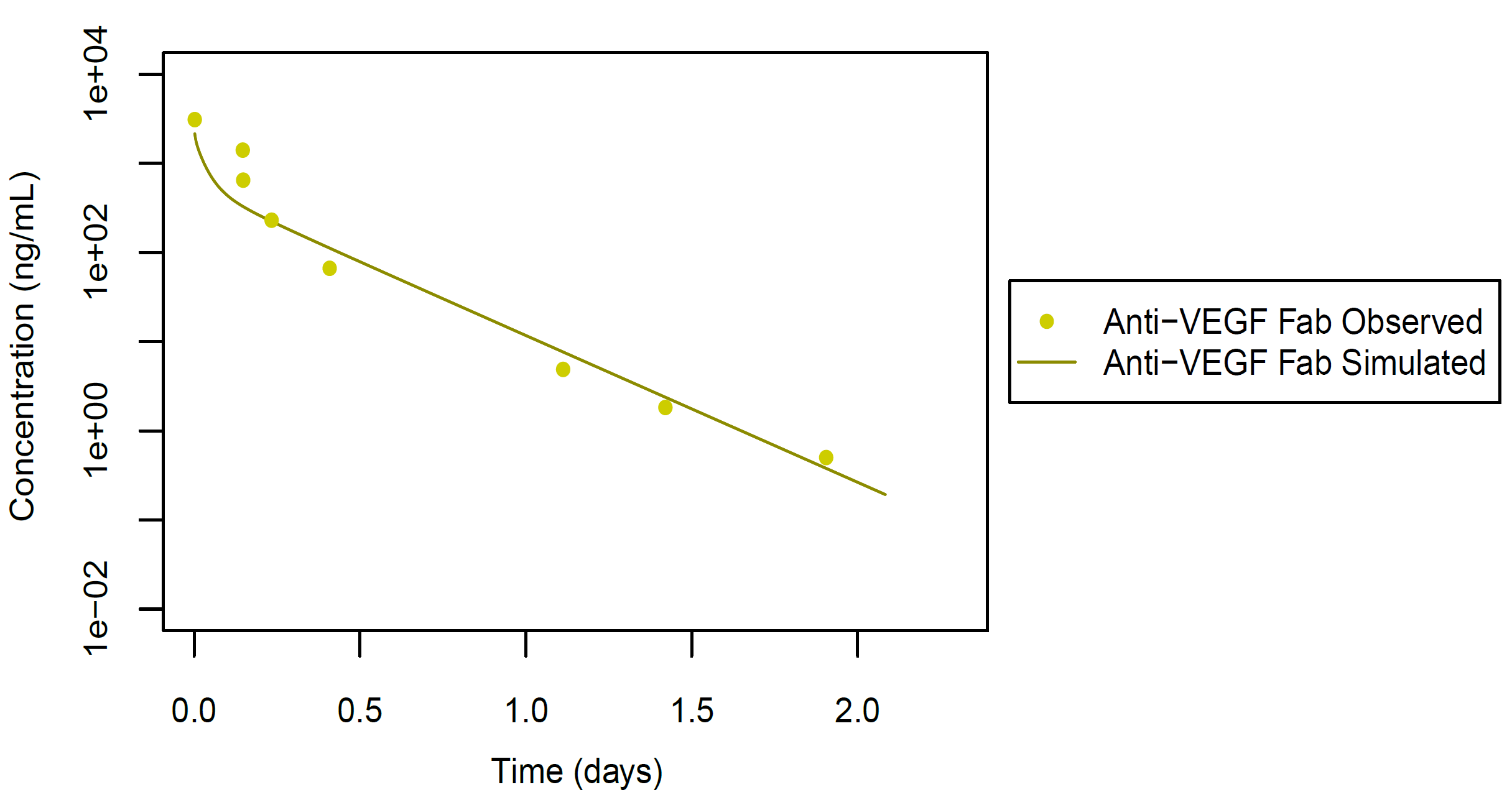


| **Parameter name** | **Optimal value** | **Start value** | **Reference** |
| --- | --- | --- | --- |
| Rh | 2.40 nm | 2.40 nm | (Hutton-Smith et al., 2016) |
| VEGF concentration | 1.15E-7 µmol/L | 0.0113 µmol/L | (Basu et al., 2020) |
| KD | 20 pmol/L | 46 pmol/L | (Papadopoulos et al., 2012) |
| k_off_ | 7.30E-6 1/sec | 7.30E-6 1/sec | (Papadopoulos et al., 2012) |
| k_deg_ | 1.13E-7 1/min | 3.34E-5 1/min | (Basu et al., 2020) |
| k_to_ | 2.78E-4 1/min | 2.78E-4 1/min | (Basu et al., 2020) |
| R_CL_ | 0.56 mL/min/kg | 0.50 mL/min/kg | (Li and Shah, 2019) |

**Supplementary Figure 1.** Observed serum concentration time profiles of Anti-VEGF Fab following intravenous bolus administration of 0.5 mg in rabbit (data from Gadkar et al., 2015). Table represents the parameters used for model building in PK-Sim^®^ and MoBi^®^.


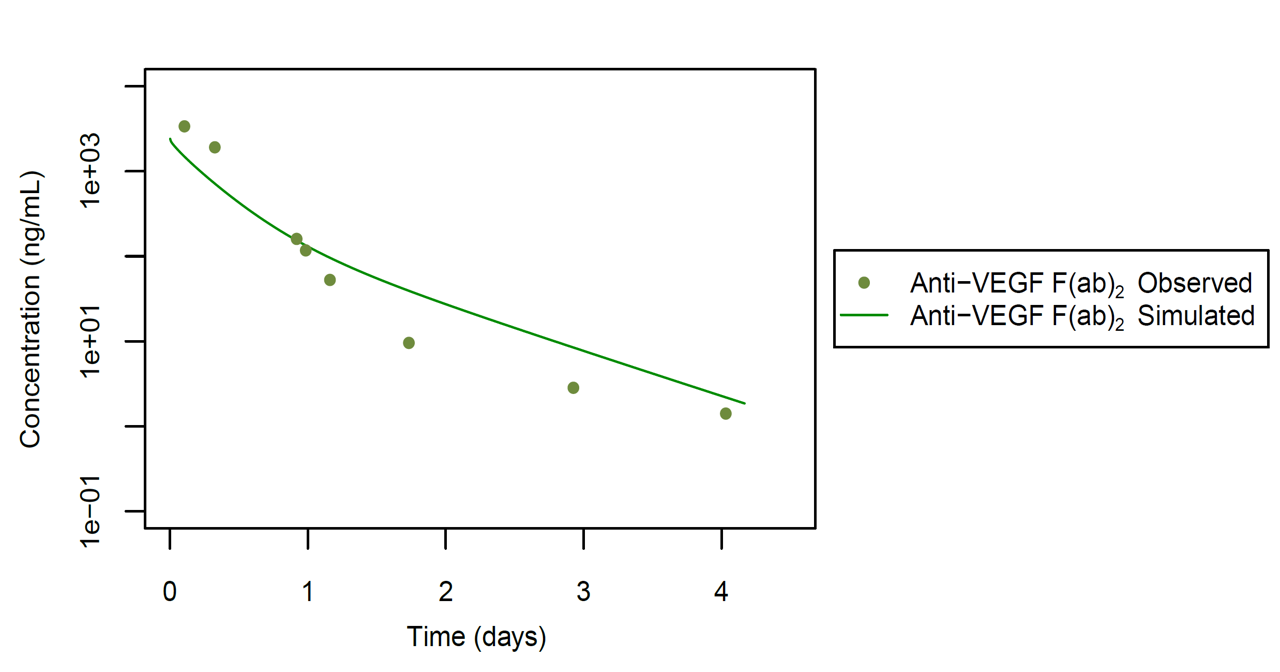


| **Parameter name** | **Optimal value** | **Start value** | **Reference** |
| --- | --- | --- | --- |
| Rh | 3.70 nm | 3.07 nm | (Hutton-Smith et al., 2016) |
| VEGF concentration | 1.15E-7 µmol/L | 1.15E-7 µmol/L | estimated^$^ |
| KD | 0.49 pmol/L | 0.49 pmol/L | (Papadopoulos et al., 2012) |
| k_off_ | 0.19 1/sec | 2.01E-5 1/sec | (Papadopoulos et al., 2012) |
| k_deg_ | 0.98 1/min | 3.34E-5 1/min | (Basu et al., 2020) |
| k_to_ | 2.78E-4 1/min | 2.78E-4 1/min | (Basu et al., 2020) |
| R_CL_ | 0.10 mL/min/kg | 0.50 mL/min/kg | (Li and Shah, 2019) |

**Supplementary Figure 2.** Observed serum concentration time profiles of Anti-VEGF F(ab)_2_ following intravenous bolus administration at 0.5 mg in rabbit (data from Gadkar et al., 2015). Table represents the parameters used for model building in PK-Sim^®^ and MoBi^®^, ^$^ estimated values consistent with earlier simulations.


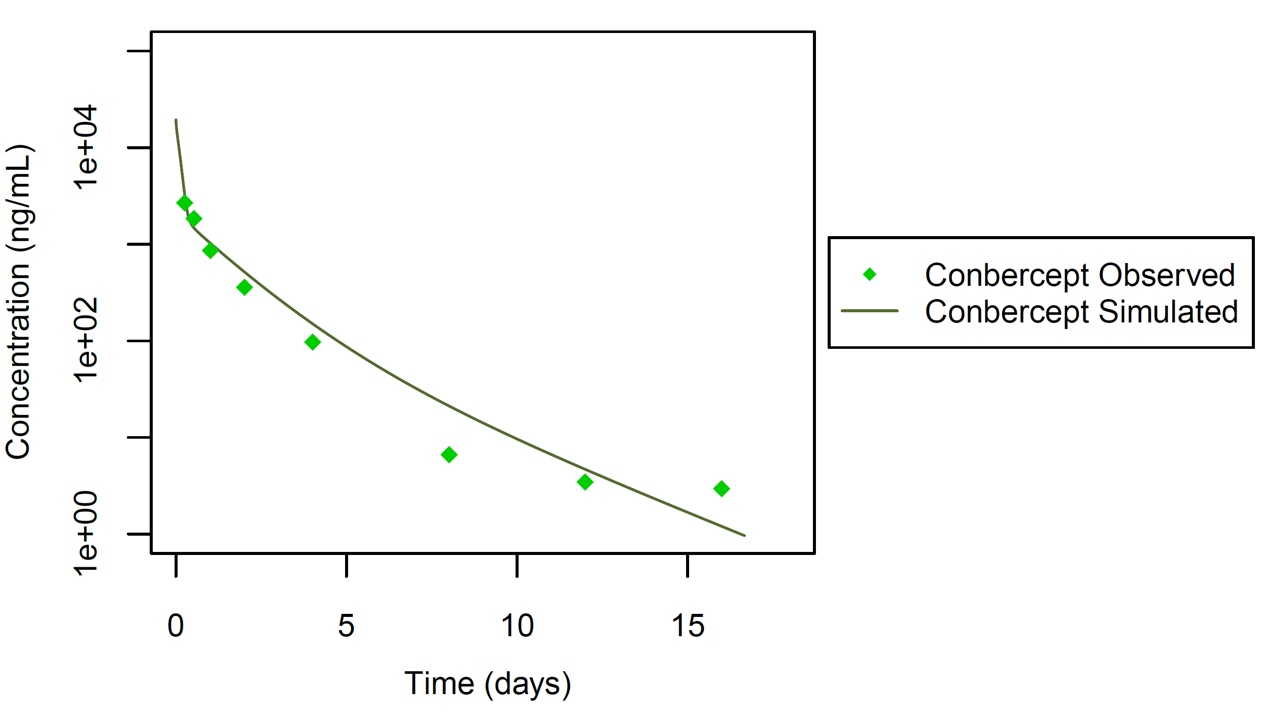


| **Parameter name** | **Optimal value** | **Start value** | **Reference** |
| --- | --- | --- | --- |
| KD-FcRn | 2.59 µmol/L | 2.59 µmol/L | estimated^$^ |
| Rh | 3.00 nm | 3.51 nm | (Hutton-Smith et al., 2016) |
| VEGF concentration | 1.15E-7 µmol/L | 1.15E-7 µmol/L | estimated^$^ |
| KD-VEGF | 1.53 pmol/L | 58 pmol/L | (Papadopoulos et al., 2012) |
| k_off_ -VEGF | 0.02 1/sec | 3.10E-5 1/sec | (Papadopoulos et al., 2012) |
| k_deg_ -VEGF | 2.22E-4 1/min | 3.34E-5 1/min | (Basu et al., 2020) |
| k_to_ -VEGF | 2.78E-4 1/min | 2.78E-4 1/min | (Basu et al., 2020) |
| PIGF concentration | 1.15E-7 µmol/L | 1.15E-7 µmol/L | estimated^$^ (Zhou et al., 2014) |
| KD-PIGF | 1.87E-3 pmol/L | 58 pmol/L | (Papadopoulos et al., 2012) |
| k_off_ -PIGF | 0.02 1/sec | 3.10E-5 1/sec | (Papadopoulos et al., 2012) |
| k_deg_ -PIGF | 614.6 1/min | 3.34E-5 1/min | (Basu et al., 2020) |
| k_to_ -PIGF | 2.03E+04 1/min | 2.78E-4 1/min | (Basu et al., 2020) |

**Supplementary Figure 3.** Observed serum concentration time profiles of Fc fusion protein conbercept following intravenous bolus administration at 3.0 mg in rabbit (data from Li et al., 2012). Table represents the parameters used for model building in PK-Sim^®^ and MoBi^®^, ^$^ estimated values consistent with earlier simulations.

Basu, S., Lien, Y.T.K., Vozmediano, V., Schlender, J.F., Eissing, T., Schmidt, S., et al. (2020). Physiologically Based Pharmacokinetic Modeling of Monoclonal Antibodies in Pediatric Populations Using PK-Sim. *Front Pharmacol* 11**,** 868. doi: 10.3389/fphar.2020.00868.

Hutton-Smith, L.A., Gaffney, E.A., Byrne, H.M., Maini, P.K., Schwab, D., and Mazer, N.A. (2016). A Mechanistic Model of the Intravitreal Pharmacokinetics of Large Molecules and the Pharmacodynamic Suppression of Ocular Vascular Endothelial Growth Factor Levels by Ranibizumab in Patients with Neovascular Age-Related Macular Degeneration. *Mol Pharm* 13(9)**,** 2941-2950. doi: 10.1021/acs.molpharmaceut.5b00849.

Li, Z., and Shah, D.K. (2019). Two-pore physiologically based pharmacokinetic model with de novo derived parameters for predicting plasma PK of different size protein therapeutics. *J Pharmacokinet Pharmacodyn* 46(3)**,** 305-318. doi: 10.1007/s10928-019-09639-2.

Papadopoulos, N., Martin, J., Ruan, Q., Rafique, A., Rosconi, M.P., Shi, E., et al. (2012). Binding and neutralization of vascular endothelial growth factor (VEGF) and related ligands by VEGF Trap, ranibizumab and bevacizumab. *Angiogenesis* 15(2)**,** 171-185. doi: 10.1007/s10456-011-9249-6.

Zhou, L., Lu, G., Shen, L., Wang, L., and Wang, M. (2014). Serum levels of three angiogenic factors in systemic lupus erythematosus and their clinical significance. *Biomed Res Int* 2014**,** 627126. doi: 10.1155/2014/627126.
